# Cell shape controls rheotaxis in small parasitic bacteria

**DOI:** 10.1371/journal.ppat.1010648

**Published:** 2022-07-14

**Authors:** Daisuke Nakane, Yoshiki Kabata, Takayuki Nishizaka

**Affiliations:** 1 Department of Engineering Science, Graduate School of Informatics and Engineering, The University of Electro-Communications, Tokyo, Japan; 2 Department of Physics, Gakushuin University, Tokyo, Japan; Miami University, UNITED STATES

## Abstract

Mycoplasmas, a group of small parasitic bacteria, adhere to and move across host cell surfaces. The role of motility across host cell surfaces in pathogenesis remains unclear. Here, we used optical microscopy to visualize rheotactic behavior in three phylogenetically distant species of *Mycoplasma* using a microfluidic chamber that enabled the application of precisely controlled fluid flow. We show that directional movements against fluid flow occur synchronously with the polarized cell orienting itself to be parallel against the direction of flow. Analysis of depolarized cells revealed that morphology itself functions as a sensor to recognize rheological properties that mimic those found on host-cell surfaces. These results demonstrate the vital role of cell morphology and motility in responding to mechanical forces encountered in the native environment.

## Author summary

The small, parasitic bacterium *Mycoplasma pneumoniae* attaches to, and moves over, host cell surfaces. Adherence to host surfaces and motility are critical for the pathogenicity of *M*. *pneumoniae*. The role of motility by *M*. *pneumoniae in vivo*, however, is poorly understood. Host airways generate constant fluid flow toward the mouth as part of their defense against pathogens and irritants. Consequently, pulmonary invaders must counter the rheological forces found in host airways in order to successfully colonize the host. Here, we demonstrate that *M*. *pneumoniae* exhibits directional movement against fluid flow. These findings suggest there is a vital role for rheotactic motility that has evolved in order to overcome host defense mechanisms such as mucociliary clearance.

## Introduction

Mycoplasmas are small parasitic bacteria with small genomes that lack a cell wall [1]. These relatively simple and small species lack the surface appendages important for host colonization found in other bacterial pathogens, such as flagella or pili. Some species of *Mycoplasma*, however, exhibit a type of motility known as gliding motility [2,3]. Currently, over ten *Mycoplasma* spp. have been shown to form a unique protrusion of the cell membrane [4–10] where the molecular machinery required for gliding motility is localized, which results in a characteristic polarized cell shape. *Mycoplasmas* move over surfaces along the axis of cell polarity, with the membrane protrusion in the lead [8,9]. Unlike many other motile species of bacteria, *Mycoplasmas* do not possess two-component-signaling systems, and therefore do not exhibit chemotaxis [11,12], raising the question of how motile *Mycoplasma* spp. reach their target environments.

*Mycoplasma pneumoniae* causes human respiratory tract infections and is responsible for approximately 4% to 8% of community-acquired bacterial pneumonia [13]. Adherence of *M*. *pneumoniae* to host cells accompanied by gliding motility requires a membrane protrusion called an attachment organelle (AO) [14,15]. While *M*. *pneumoniae* forms biofilms on host surfaces, which is presumed to aid in evading the host immune system [16], gliding motility has only a limited role in biofilm formation *in vitro* [17]. There is, however, a primary innate defense system in the human airway termed mucociliary clearance, where the cilia of epithelial cells generate surface flow of mucous to transport foreign pathogens toward the mouth [18].

An open question in microbial biology has been how the mechanical forces microbes encounter in their environmental niches affect their ability to persist in and colonize those niches [19]. One well documented behavior that many micro-sized swimmers exhibit is a reorientation of the cell body relative to the direction of flow and subsequent migration upstream. This style of swimming behavior in response to flow is called rheotaxis, and has been observed in sperm [20], bacteria [21–23], and artificial cells [24]. Rheotaxis is commonplace in higher organisms such as fish [25,26]. The first report of rheotaxis in prokaryotes was over 30 years ago in the fish pathogen *Mycoplasma mobile*, which migrates to the gills of fish by employing a type of motility known as gliding motility [27,28]. The means by which *M*. *mobile* successfully navigates its way to the gills, however, has remained a mystery.

In this report, we show that *M*. *pneumoniae* exhibits positive rheotactic behavior. We also demonstrate that two other, phylogenetically distant mycoplasma species, *M*. *mobile* and *M*. *penetrans*, employ the same style of rheotactic gliding motility as *M*. *pneumoniae*, suggesting that this behavior is widespread in the *Mycoplasma* genus. Furthermore, we demonstrate that the cell axis is oriented parallel to the direction of flow, with the attachment organelle at the leading pole, enabling *M*. *pneumoniae* to navigate upstream against fluid flow. These results provide direct evidence that the gliding motility of mycoplasmas is a mechanical response that recognizes flow direction, and that this response may have a vital role in the infection process.

## Results

### Rheotaxis in *M*. *pneumoniae*

**Fig 1:**
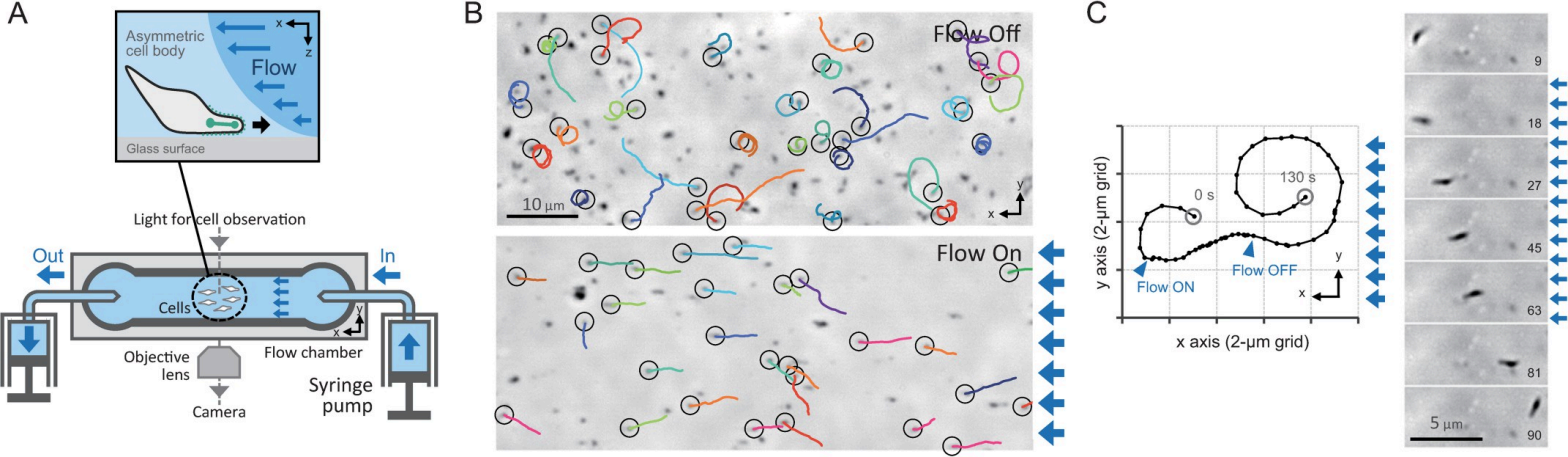
Rheotactic behavior of *M*. *pneumoniae*. (A) Experimental setup. Cells were poured into a flow chamber connected to a syringe pump. Precisely controlled fluid flow was applied from the right side in the following images, and the cell behaviors were observed under phase-contrast microscopy. *Left upper*: Schematic of positive rheotactic behavior. A cell adheres to the glass surface at the attachment organelle and moves over the surface in an upstream direction against the fluid flow. (B) Field image of the rheotactic behavior. Cell trajectories for 40 s (color lines) are plotted on the phase-contrast images. The start position of a trajectory is marked by the black circle. The blue arrows on the right side of the image represent the direction of the fluid flow. *Upper*: No fluid flow. *Lower*: Fluid flow at 1.7 mm/s. (C) Single-cell trajectory. *Left*: Moving trace at 2-s intervals. Arrowheads indicate cell positions at the start and the end of fluid flow. *Right*: Time-lapsed image. Each time point is presented at the bottom right of the image.

We constructed a flow chamber to observe gliding motility under fluid flow (Fig 1A). The chamber was connected to a syringe pump in order to apply directional flow at a precisely defined flow rate. Flow speed in the chamber was measured by measuring the drift velocity of *M*. *pneumoniae* M129 cells that occasionally detached from the glass surface of the chamber when flow was applied. Using this method, we confirmed that flow speed had a linear relationship with the flow rate of the syringe pump (S1 Fig). Flow speed was controllable up to 15 mm/s, which is 3,000 times faster than the gliding speed of *M*. *pneumoniae* [6].

We observed that M129 cells moved with curved trajectories without flow in the chamber, whereas cells moved upstream in straight trajectories 10 s after applying a flow speed of 1 mm/s (Fig 1B and S1 Movie). The cells returned to curved trajectories when the syringe pump was switched off. Notably, flow affected the orientation of the cell body such that the longer cell axis was aligned parallel with the direction of flow (Fig 1C). Hereafter, we refer to upstream-directed motility as positive rheotaxis.

Positive rheotactic behavior was observed for over 80% of gliding *M*. *pneumoniae* cells (*N* = 50 cells). In our experimental setup, we observed cells at 30°C and not at the optimal growth temperature of 37°C. This lower temperature minimized thermal drift during the acquisition of cell images. Although the gliding speed decreased to 0.18 ± 0.04 μm/s at 30°C, which is 50% slower than that at 37°C, the cells still showed clear and smooth gliding motility (S2 Fig). All experiments with *M*. *pneumoniae* were performed at 30°C unless otherwise stated.

**Fig 2:**
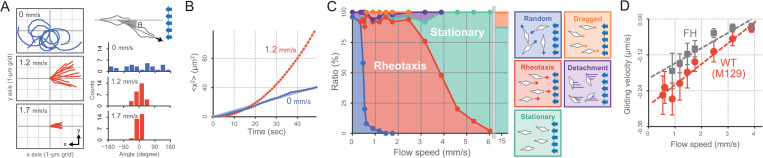
Effect of flow speed on gliding motility of *M*. *pneumoniae*. (A) *Left*: Moving traces of cells at flow speeds of 0, 1.2, and 1.7 mm/s. The traces for 10 s from 10 cells are overlayed. The cell position at time 0 is plotted at the center of the graph. *Right*: Distribution of the angle between moving direction and the flow axis (*N* = 30), which is schematically illustrated at the top. The cell moving against the fluid flow was defined as the zero angle. (B) MSD plots. The time course of mean square displacement (MSD) from 30 cells along the flow direction is plotted. *Red*: Flow speed at 1.2 mm/s. *Blue*: No fluid flow. Red and blue lines represent hyperbolic and liner fitting, respectively. (C) Fraction of cell motility pattern. The motility pattern under various flow speeds was categorized into five groups, as presented on the left. (D) Relationship between cell gliding velocity and flow speed. The average and standard deviation (SD) of cell gliding velocity during positive rheotaxis were plotted at each flow speed. Red: Type 1 strain M129 as standard in this study (*N* = 30). Gray: Type 2 strain FH (*N* = 15). Dashed lines present linear approximations.

We next examined the dependency of cell movement on flow speed to gain further insight into positive rheotactic behavior by *M*. *pneumoniae* (Fig 2A). We found that at flow speeds greater than 0.5 mm/s, cells changed their previously random orientations to adopt orientations parallel to the direction of flow. During positive rheotaxis, the angle of the moving direction relative to the flow axis was 0°, on average. The cell trajectories of rheotaxing cells were observed to be straight with no bias, while that those in no-flow conditions have been reported to be biased to the right (clockwise when viewed from above) [29]. In agreement with previous observations, we found that the mean square displacement (MSD) of M129 cells increased linearly over time in the absence of flow, demonstrating that gliding motility *sans* flow is random with an apparent diffusion coefficient of 0.2 μm^2^/s (Fig 2B, *Blue*). In contrast, the MSD of cells at a flow speed of 1.2 μm/s had a parabolic line, indicating that gliding motility is directional under flow (Fig 2B, *Red*).

Positive rheotactic behavior was found to stop at flow speeds >4.0 mm/s, but orientation of the long axis of the cell parallel to the direction of flow persisted (Fig 2C). These stationary cells did not detach even at 15 mm/s, the fastest flow speed in our experimental setup. Some cells under flow were categorized as having different motility patterns than the majority of cells, such as detachment from the surface or being dragged backward, but the proportion of these two motility patterns combined was less than 20% of the cell population. We report cell displacement in the direction of flow as negative values in our measurements.

Positive-rheotactic gliding velocity showed a positive correlation as a function of the flow speed over the range of 0.5–3.8 mm/s, which was linearly extrapolated to zero to give a flow speed of 4.1 mm/s (Fig 2D). Clinical isolates of *M*. *pneumoniae* can be classified into two major groups, with strain M129 a representative of type 1 isolates, and another strain, FH, representative of type 2 isolates [30]. The positive correlation between the flow speed and cell velocity during positive rheotaxis was also observed in FH (Fig 2D and S2 Movie).

To determine whether there is a link between rheotaxis and gliding motility, we observed the behavior of mutants of *M*. *pneumoniae* M129 possessing AOs of different sizes (S3 Fig). The dec_5 and inc_5 mutants, harboring size-modified HMW2 derivatives, form longer and shorter AOs, respectively [31]. We found that in both of these backgrounds, cells showed positive rheotactic behavior. Gliding velocity showed a positive correlation as a function of flow speed over a range of 0.8–1.7 mm/s. However, extrapolation of gliding speed at zero to the flow velocity was slower than that from WT (S3 and 2D Figs). We speculate that this could be caused by impaired binding of the attachment organelle to the glass surface in size-modified HMW2 mutants [31].

**Fig 3:**
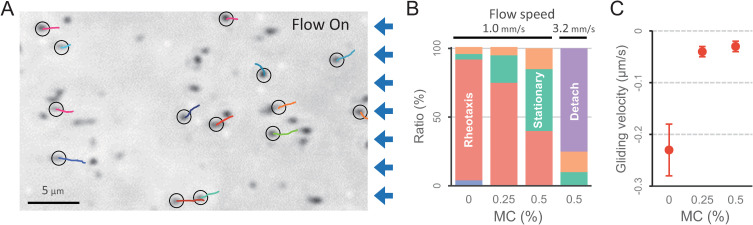
Rheotaxis of *M*. *pneumoniae* in a viscous environment. (A) Field image of cell behavior in the presence of 0.25% methylcellulose (MC). Fluid flow at 1 mm/s was applied. Cell trajectories for 40 s (color lines) were plotted on the phase-contrast images. The start position of a trajectory is marked by the black circle. The blue arrows on the right side of the image represent the direction of the fluid flow. (B) Fraction of cell motility pattern. The motility pattern under fluid flow was categorized into five groups at each MC concentration (*N* = 30). The color code and the definitions are the same as those presented in Fig 2B. (C) Cell gliding velocity. The average and SD of cell gliding velocity during positive rheotaxis are shown (*N* = 30).

To determine whether *M*. *pneumoniae* can counter the force of airway-cilia-generated mucus flow, we also analyzed rheotaxis in viscous environments (Fig 3A and S3 Movie). We added methylcellulose (MC) at concentrations of 0.25%–0.5% to increase viscosity of the fluid in the flow chamber. We found that viscous-fluid flow had a large effect on the gliding-motility speed of cells, relative to non-viscous-fluid flow. Half of the cell population was stationary at a flow speed of 1.0 mm/s, and most cells detached from the glass surface at a flow speed of 3.2 mm/s in the presence of 0.5% MC (Fig 3B and 3C and S4 Movie).

### Cell shape affects Rheotaxis

**Fig 4:**
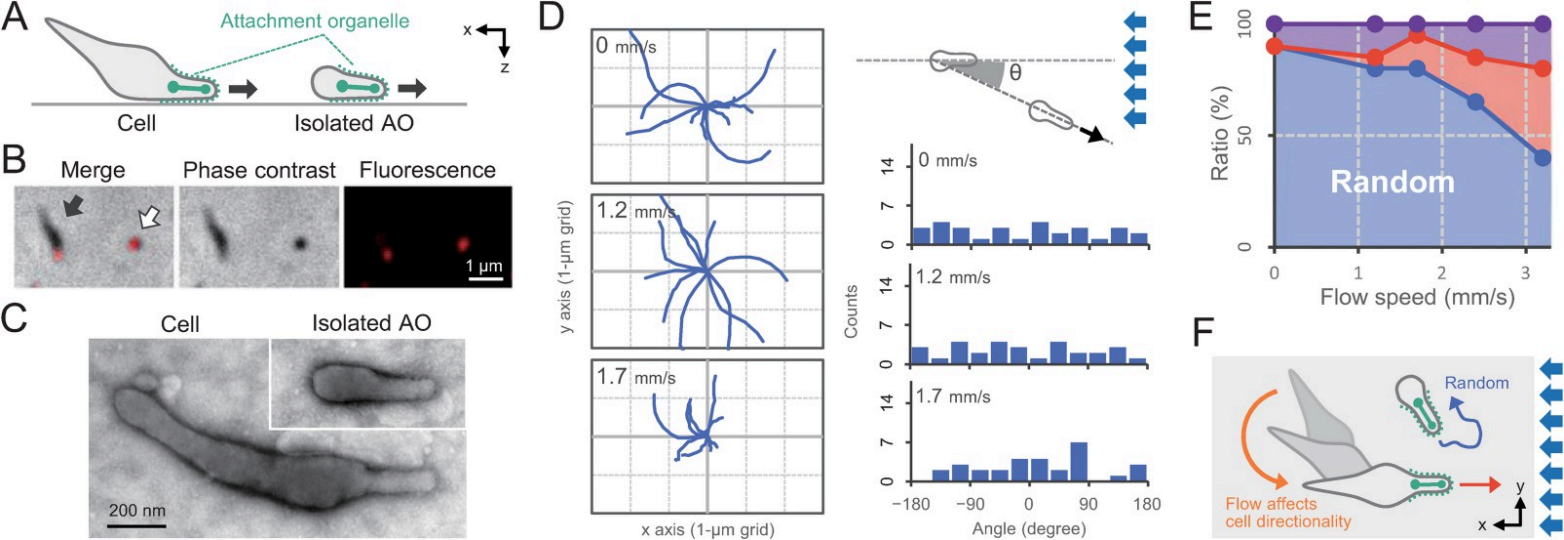
Effect of fluid flow on the motility pattern of the isolated attachment organelle (AO) in *M*. *pneumoniae*. (A) Schematic of the intact cell and the isolated AO. (B) Localization of the AO. P1 protein was immunofluorescently labeled by a monoclonal antibody [61]. Phase-contrast, fluorescence, and merged images of cells are presented. *Black arrow*: Intact cell. *White arrow*: Isolated AO. (C) EM image of an intact cell and an isolated AO. Cells were chemically fixed and observed by negatively stained EM. (D) *Left*: Moving trace of the cell at flow speeds of 0, 1.2, and 1.7 mm/s. The traces for 10 s from 10 cells were overlayed. The cell position at time 0 was plotted at the center of the graph. *Right*: Distribution of the angle between the moving direction and flow axis (*N* = 30), schematically illustrated at the top. A cell moving against the fluid flow was defined as the zero angle. (E) Fraction of cell motility pattern. The motility pattern under fluid flow was categorized into five groups, as presented in Fig 2B. (F) Schematic of the rheotactic behavior in response to a fluid flow. The flow affected the directionality of intact cells, which aligned along the flow direction with AO as the leading pole. In contrast, the isolated AO shows more random movement because of the absence of the cell body.

Over the course of our experiments, we often observed small cellular particles exhibiting gliding motility in the flow chamber. These particles exhibited random gliding motility under flow (*i*.*e*. rheotaxis was not observed) (S5 Movie). Intact cells adhere to the glass surface at the leading end of the cell where the AO is localized. The small cellular particles we observed appeared to be composed of a cell fragment containing the AO, but lacking the rest of the cell body (Fig 4A). Immunofluorescent microscopy against the P1 protein, a major surface component of the AO (Fig 4B) and transmission electron microscopy (TEM) confirmed that the small cellular particle was the leading end of the cell that had detached from the rest of the cell body (Fig 4C). Previous work has reported that some mutants of *M*. *pneumoniae* produce isolated AOs, which lack DNA and continue to glide for up to 30 minutes after separating from an intact cell [32].

To study gliding motility and rheotaxis of detached but otherwise WT AOs, we applied shear force to a WT cell suspension, which produced numerous detached AOs for observation. The presence of isolated AOs was confirmed by their small size and smooth movement over the glass surface, as well as a gliding velocity ~1/20^th^ that of the intact cells. The isolated AOs exhibited gliding motility with curved trajectories even at a flow speed of 1.7 mm/s (Fig 4D) and a reduced dependence on flow speed to stimulate rheotaxis compared to intact cells (Fig 4E). This suggests that polar cell shape and asymmetric cell binding are required for recognition of flow direction during rheotaxis (Fig 4F).

### Rheotaxis in *M*. *mobile* and *M*. *penetrans*

**Fig 5:**
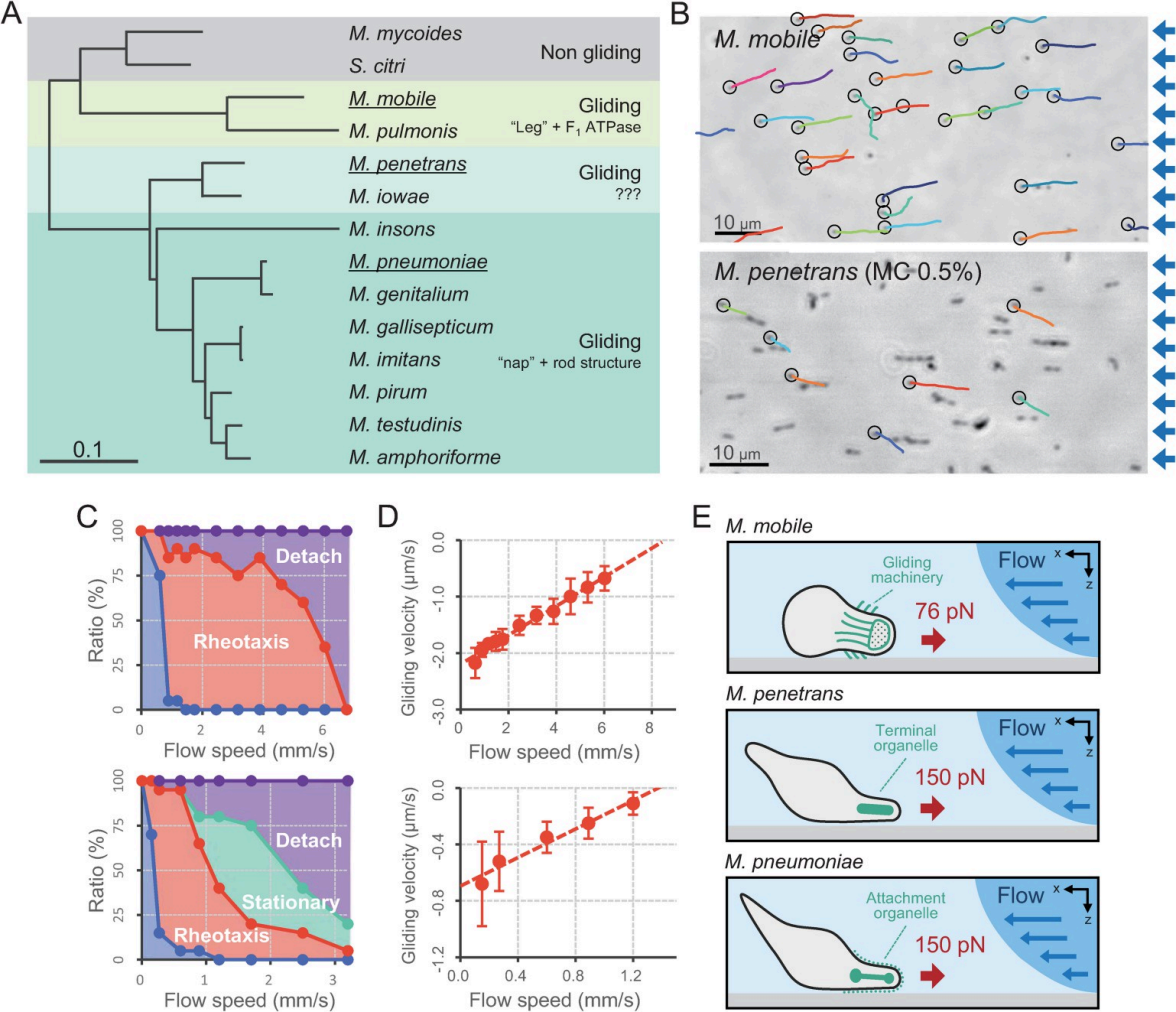
Rheotactic behaviors in gliding *Mycoplasma*. (A) Phylogenetic tree based on the sequences of the 16S ribosomal RNA. Twelve gliding species and two non-gliding species in Mollicutes are presented. Gliding mycoplasmas were categorized into three types based on external and internal structural features responsible for adherence and gliding motility [62]. The scale represents the number of substitutions per site. The species used in this study are underlined. (B) Cell trajectories. The trajectories (color lines) of *M*. *mobile* for 6 s and *M*. *penetrans* for 15 s were overlayed to each cell image under phase-contrast microscopy. Fluid flow was applied from the right side of the image at 1.7 mm/s for *M*. *mobile* and 0.6 mm/s for *M*. *penetrans*. The cells of *M*. *penetrans* were observed in the presence of 0.5% methylcellulose. The start position of a trajectory is marked by the black circle. The blue arrows on the right side of the image represent the direction of the fluid flow. (C) Fraction of cell motility pattern. The motility pattern under fluid flow was categorized into five groups, as presented in Fig 2B. *Upper*: *M*. *mobile*. *Lower*: *M*. *penetrans*. (D) Relationship between the cell gliding velocity and flow speed. The average and SD of the gliding velocity during positive rheotaxis are plotted for each flow speed (*N* = 15). The dashed lines present linear approximations. *Upper*: *M*. *mobile*. *Lower*: *M*. *penetrans*. (E) Schematic of rheotaxis in three gliding mycoplasmas. The estimated maximum forces, the polar cell shapes, and the structures responsible for adherence and gliding motility are presented. *Top*: *M*. *mobile*. *Middle*: *M*. *penetrans*. *Bottom*: *M*. *pneumoniae*.

We next examined the relationship between motility and flow speed in *M*. *mobile* and *M*. *penetrans*, phylogenetically distant species from *M*. *pneumoniae*, in order to generalize our findings (Fig 5A) [4,10]. We found that *M*. *mobile* showed curved trajectories without flow in the sample chamber, as previously reported [29], but that cells assumed straight, upstream trajectories at a flow speed of 1 mm/s (Fig 5B *upper* and S6 Movie). In contrast, *M*. *penetrans* did not attach to the glass surface under the same conditions, which is consistent with a previous study which reported that gliding motility by *M*. *penetrans* requires supplementation of the media with 3% gelatin [7]. To mimic the addition of gelatin, we increased the fluid viscosity by adding 0.5% MC and found that *M*. *penetrans* exhibited smooth gliding motility under these conditions (S7 Movie). Gliding speed of *M*. *penetrans* in 0.5% MC under flow was 2.19 ± 0.42 μm/s at an optimal growth temperature of 37°C and decreased to 0.65 ± 0.28 μm/s at room temperature (S4A Fig). Cell trajectories were curved and unidirectional, keeping the membrane protrusion as the leading end (S4B Fig), where the terminal organelle has been suggested to be localized (S4C Fig) [7]. These curved trajectories changed to straight when we applied a fluid flow of 0.6 mm/s (Fig 5B *lower* and S8 Movie). We observed a motility pattern by *M*. *penetrans* that shifted from random under no flow, to positive rheotaxis, to stationary, and finally to detachment from the glass surface as the flow speed increased (Fig 5C *lower*).

Unlike *M*. *pneumoniae* or *M*. *penetrans*, cells of *M*. *mobile* did not stop gliding motility at higher flow speeds (Fig 5C *upper*). We plotted the velocity of gliding motility as a function of the flow speed measured at a temperature of 25°C. This revealed a positive correlation, which was linearly extrapolated to zero-gliding velocity to give flow speeds of 8.4 mm/s and 1.3 mm/s in *M*. *mobile* and *M*. *penetrans*, respectively (Fig 5D).

## Discussion

In this report, we directly visualized positive rheotaxis by *M*. *pneumoniae* and revealed that the directionality of rheotaxis is controlled by polar cell shape (Figs 1, 2, and 4). This is consistent with previous work which has shown that upstream motion in other microorganisms is promoted by asymmetric cell shape or asymmetric binding [20,22,33]. By closely examining cell movement in *M*. *pneumoniae*, we found that, under fluid flow, the rear part of the cell body rotates around the attachment organelle, enabling the long axis of the cell to align parallel to the flow axis to minimize fluid resistance. This alignment appears to be a simple mechanical response where the cell body acts as an antenna to recognize flow direction, similar to a weathervane (Fig 4F).

Polar cell shape and asymmetric cell binding are conserved in almost all gliding mycoplasmas [5,7,9,34], suggesting that this framework of passive control of rheotaxis also applies to phylogenetically distant species, such as *M*. *mobile* and *M*. *penetrans* (Fig 5E) [11,12,35]. As the molecular basis of gliding motility has no obvious similarity among these species, unidirectional gliding motility may have arisen via convergent evolution to enable rheotactic behavior in response to external mechanical signals.

*M*. *pneumoniae* cells exhibited rheotaxis in highly viscous environments at flow speeds up to 3.2 mm/s (Fig 3). By approximating cell shape as a cylinder, we estimated the maximum force exerted during gliding motility to be 150 pN (S5 Fig), which is higher than the stall force in *M*. *mobile* [28,36]. Thus, the gliding force of a single *M*. *pneumoniae* cell is sufficient to counter that of human airway cilia at 60 pN [37], indicating that cells are not swept away by mucociliary clearance.

Many biological interactions are enhanced by load-dependent mechanisms called catch bonds, which have been well-studied in bacteria [38], and have recently also been suggested to exist in *M*. *mobile* [39]. The adherence of *M*. *pneumoniae* and *M*. *mobile* is postulated to involve surface proteins [40,41] that bind sialic acid targets on the host cells [42]. These surface proteins may possess rheological properties in the actual host-surface environment.

We hypothesized that rheotaxis might play a critical role for pathogenicity in gliding mycoplasmas. Once *M*. *pneumoniae* cells have been transmitted to the host airway through aerosols [13], the unidirectional flow of the mucociliary defense system of the host enables *M*. *pneumoniae* to navigate to the lungs, opposite the direction of mucociliary flow. Here, we showed that cells exhibit rheotaxis even at a flow speed of 1.0 mm/s in the presence of 0.5% MC (Fig 3). This experimental flow speed is much greater than that of *in vivo* mucociliary transport in a newborn pig model system, which was reported as 0.1 mm/s [43].

The flow-induced positive rheotaxis we observed in this study might be applicable to other gliding mycoplasmas. *M*. *mobile* was isolated from the gills of a tench (*Tinaca tinca*) [44], around whose gills the fluid flow speed was reported to be 7.6 mm/s [45], which has good agreement with our measurement of the maximum flow speed at which *M*. *mobile* remained adhered in the flow chamber (Fig 5). *M*. *penetrans* was originally isolated from the urinary tract [46], where ureter peristalsis drives the flow of urine from the kidneys to the bladder at speeds that reach approximately 20–30 mm/s [47]. Our measurement suggests that *M*. *penetrans* cells can adhere at a flow speed of 36 mm/s in low-viscosity fluids such as urine (Fig 5). Additionally, the maximum force exerted during gliding motility by *M*. *penetrans* is higher than that of type I pili in uropathogenic *Escherichia coli* at 60 pN, suggesting that the force of gliding motility in *M*. *penetrans* is sufficient to persist in the urinary tract [48].

The measurements in this study were taken from experiments in a simplified flow chamber, where the temperature is controllable up to 30°C. The flow inside the chamber is typically laminar, and flow speed decreases at the boundary of solid/liquid interface [49]. Consequently, further studies are required to verify our observations of positive rheotaxis in a more realistic environment, *e*.*g*., through the use of microfluidic devices with complex shapes or surfaces that resemble the cilia of host cells. Nevertheless, the positive rheotactic behavior observed here sheds light on the physiological relevance of cell motility in a minimal life form.

## Materials and methods

### Bacterial growth conditions

*M*. *pneumoniae* M129 (subtype 1) and FH (subtype 2) [50] were grown in PPLO medium at 37°C in tissue culture flasks. *M*. *pneumoniae* M129 was used as the wild-type (WT) strain. For selection and maintenance of *M*. *pneumoniae* antibiotic-resistant strains, gentamicin and chloramphenicol were used at 18 and 15 μg ml^–1^ for inc_5 and dec_5 mutants, respectively [31]. *M*. *mobile* gli521(P476R), an adhesive mutant [51], was grown in Aluotto medium at 25°C in a tissue culture flask to an OD_600_ of 0.1–0.2. *M*. *penetrans* GTU-54-6A1 (ATCC55252) was grown in SP4 broth at 37°C in a tissue culture flask to an OD_600_ of 0.2–0.5 [7,52].

### Cell preparation for gliding motility

*M*. *pneumoniae* cells were prepared under optimized conditions for gliding motility, as described previously [31]. The tissue culture flask where *M*. *pneumoniae* cells were grown on the bottom was washed twice with PBS/HS, which was composed of 10% horse serum (HS) (Gibco) in phosphate-buffered saline (PBS) of 75 mM sodium phosphate (pH 7.4) and 68 mM NaCl. The cells that remained attached to the bottom of the tissue culture flask were scraped into PBS/HS. The HS used in the preparation of the flow chamber was used without heat inactivation [6,31]. The cell suspension was passed through a syringe needle > three times (21G × 38 mm; Terumo) and filtered with a syringe-driven filter unit (Millex LH 0.45 μm; Millipore) [53,54]. The syringe passaged and filtered cell suspension was subsequently used for flow experiments. For the preparation of the isolated AO, the suspension was passed through the syringe needle >15 times and filtered with a syringe-driven filter unit.

For observation of *M*. *pneumoniae* in a viscous environment, cells were scraped into either 0.25% or 0.5% MC (M0512, 4,000 cP at 2% solution; Sigma-Aldrich) with 10% HS in PBS and used for flow experiments.

For observation of *M*. *mobile*, the cell suspensions were centrifuged at 12,000 × *g* for 4 min, and the pellet was suspended in PBS/HS at one-tenth of the original culture volume, and subsequently used for flow experiments. For *M*. *penetrans*, the cell suspension was centrifuged at 12,000 × *g* for 4 min. The resulting pellet was suspended in PBS/HS containing 10% fetal bovine serum and 0.5% MC in PBS at one-tenth of the original culture volume and used for flow experiments.

### Optical microscopy and data analyses

All measurements were performed under an inverted microscope (IX71; Olympus) equipped with an objective lens (LUCPLFLN 40×PH, N.A. 0.6, LUCPLFLN 60×PH, N.A. 0.7 and UPLANSAPO 100×PH, N.A. 1.4; Olympus), a CMOS camera (Zyla 4.2; Andor, DMK33U174; Imaging Source, and FASTCAM Mini AX; Photoron), and an optical table (HAX-0806; JVI). The microscope was kept at 30°C by heating the room for the observation of *M*. *pneumoniae* cells, but this heating was not applied for *M*. *penetrans* and *M*. *mobile*. The microscope stage was heated with a thermoplate (TP-110R-100; Tokai Hit) for examining the temperature dependency of gliding speed. Projection of the image to the camera was acquired with imaging software that came with the CMOS camera and converted into a sequential TIFF file without any compression. All data were analyzed by ImageJ v1.48 (rsb.info.nih.gov/ij) [55] and its plugins particle tracker [56] and multitracker.

### Flow experiments

The flow chamber was assembled by taping a coverslip with a glass slide, as described previously [57,58]. Inlet and outlet ports were created by boring through the glass slide with a high-speed precision drill press equipped with a diamond-tipped bit [2.35-mm diameter, ICHINEN MTM, Japan]. The sample chamber was prepared from a glass slide, a coverslip (Matsunami), and double-sided tape (∼100-μm-thick; 3M). After assembly, the flow chamber was incubated for 60 min at 120°C to ensure tight adhesion of the slide and coverslip. Inlet and outlet ports (N-333; IDEX Health & Science) were attached with hot-melt adhesive (Goot; Taiyo Electronic IND). The total volume of the sample chambers was adjusted to 10 μL (width: 3 mm, length: 30 mm). A syringe pump (Legato 210P; Kd Scientific) was connected to the flow chamber by a connecter and tube (F-333NX and 1512L; IDEX Health & Science) and used to control the flow rate of the buffer. The flow chamber was pre-coated with PBS/HS, as required. Cell suspensions were pipetted into the chamber and left to incubate for 10 min to allow cells to bind to the glass surface. After washing with PBS/HS or PBS/FBS, the flow chamber was used for observing gliding motility. The flow rate in the sample chamber was calculated by measuring the speed of unattached cells using a high-speed camera (FASTCAM Mini AX; Photoron), as described in the optical microscopy section.

### Force estimation

For the drag coefficient of a cell as a cylindrical rod, γ = 2π*ηL* × [ln(*L*/2*r*) − 0.20]^−1^ was applied, where *r* is cell radius, *L* is cell length, and *η* is viscosity (S5 Fig) [59,60]. The *η* used was 10^−2^ and 10^−3^ Pa·s for medium with and without 0.5% MC, respectively. The force of gliding motility was estimated by *F* = γ × *v*, where *v* is the gliding speed measured by cell displacement upstream of the fluid flow. The length and the diameter of cells were taken from reported EM images [10,31,63]. The equation for force estimation is described [59,60].

### Electron microscopy

Sample preparation for negative-stain EM followed the same protocol described previously [31]. Carbon-coated EM grids were glow-discharged by a PIB-10 hydrophilic treatment device (Vacuum Device). The cell suspension was placed on the EM grid and chemically fixed by 1% (w/v) glutaraldehyde in PBS for 10 min at room temperature. After washing three times with PBS, the grid was stained with 2% ammonium molybdate (v/v). Samples were observed under a transmission electron microscope (JEM-1400, JEOL) at 100 kV. A charge-coupled device camera captured EM images.

## Supporting information

S1 Fig Calibration of flow speed in the flow chamber. The plot of the relationship between the flow rate of the syringe pump and the flow speed near the glass surface of the chamber. The flow speed was determined by measuring the movement of non-adhered cells near the glass surface that moved passively in the flow direction. The average and SD are plotted (*N* = 10).
(EPS)

S2 Fig Temperature dependency of gliding motility in *M*. *pneumoniae*. The average and SD of gliding speed were measured at each temperature (*N* = 20).
(EPS)

S3 Fig Relationship between cell gliding velocity and flow speed in the length-controlled mutants of the attachment organelle in *M*. *pneumoniae*. The average and SD of cell gliding velocity during positive rheotactic behavior were plotted in each flow speed. Gray: Type strain M129 as WT (N = 30). Red: inc_5 mutant (N = 15). Green: dec_5 mutants (N = 15). The mutants of inc_5 and dec_5 form longer and shorter attachment organelle by the size-modified HMW2 derivatives, respectively. The linear approximations were presented by the dashed lines.
(EPS)

S4 Fig Gliding motility and cell shape of *M*. *penetrans*. (A) Temperature dependency of gliding motility. The average and SD of gliding speed were measured at each temperature (*N* = 30). (B) Phase-contrast images at intervals of 15 s at 25°C. The arrows indicate the direction of gliding during the observation period. (C) EM image of the cell. *Bottom image*: Magnification of the yellow-boxed region.
(EPS)

S5 Fig Estimation of force during gliding motility. (A) Cell shape approximation. By approximating the cell chape of *M*. *pneumoniae*, *M*. *penetrans*, and *M*. *mobile* as cylinders, the force is given by the drag coefficient and flow speed. (B) Maximum force for gliding motility. Species of gliding *Mycoplasma*, flow speed, and force are listed. The flow speed was used at the value when most cells were detached from the glass surface.
(EPS)

S1 Movie Positive rheotactic behavior of *M*. *pneumoniae* strain M129 as WT. Fluid flow at 1.0 mm/s was applied at the time point of 30 s from the right side of the image. Area: 120 μm × 90 μm.
(AVI)

S2 Movie Positive rheotactic behavior of *M*. *pneumoniae* strain FH. Fluid flow at 1.7 mm/s was applied at the time point of 30 s from the right side of the image. Area: 80 μm × 60 μm.
(AVI)

S3 Movie Positive rheotactic behavior of *M*. *pneumoniae* WT in a viscous environment. Fluid flow at 1.0 mm/s was applied at the time point of 30 s from the right side of the image in the presence of 0.25% methylcellulose. Area: 80 μm × 60 μm.
(AVI)

S4 Movie Positive rheotactic behavior of *M*. *pneumoniae* WT in a viscous environment. Fluid flow at 3.2 mm/s was applied at the time point of 30 s from the right side of the image in the presence of 0.50% methylcellulose. Area: 80 μm × 60 μm.
(AVI)

S5 Movie The behavior of the detached AO in *M*. *pneumoniae*. Blue arrows marked the detached AO at the beginning. Fluid flow at 1.7 mm/s was applied during the entire movie from the right side. Area: 64 μm × 48 μm.
(AVI)

S6 Movie Positive rheotactic behavior of *M*. *mobile*. Fluid flow at 1.7 mm/s was applied at the time point of 10 s from the right side of the image. Area: 80 μm × 60 μm.
(AVI)

S7 Movie Gliding motility of *M*. *penetrans*. The cells were suspended in an SP4 medium containing 0.50% methylcellulose and observed at 37°C. Area: 120 μm × 90 μm.
(AVI)

S8 Movie Positive rheotactic behavior of *M*. *penetrans*. Fluid flow at 0.6 mm/s was applied at the time point of 10 s from the right side of the image in the presence of 0.50% methylcellulose. Area: 120 μm × 90 μm.
(AVI)
